# Comparisons of Cooking, Dietary, and Food Safety Characteristics of Food Secure and Food Insecure Sophomores at a University in Appalachia

**DOI:** 10.13023/jah.0304.08

**Published:** 2021-10-25

**Authors:** Hannah E. Boone, Melissa D. Gutschall, Alisha R. Farris, Kimberly S. Fasczewski, Donald Holbert, Laura H. McArthur

**Affiliations:** Department of Nutrition and Health Care Management, Appalachian State University, Boone NC; Department of Health and Exercise Science, Appalachian State University, Boone NC; Department of Biostatistics, East Carolina University (retired), Greenville NC; Department of Nutrition and Health Care Management, Appalachian State University, Boone NC

**Keywords:** Appalachia, college students, food security, food group consumption, cooking self-efficacy, food safety knowledge

## Abstract

**Introduction:**

Food insecurity means lacking access to adequate, nutritious, and safe food. Collegiate food insecurity rates at ten Appalachian campuses range from 22.4% to 51.8% and have been associated with unfavorable health and academic outcomes.

**Purpose:**

This study compared cooking, dietary, and food safety characteristics of food secure (FS) and food insecure (FI) sophomores at a university in Appalachia in the context of the USDA definition of food security.

**Methods:**

Data were collected using an online questionnaire. Descriptive and inferential procedures compared FS and FI sophomores (p < 0.05).

**Results:**

Participants (n = 226) were 65.0% females, 76.1% whites, and 46% FI. About 40% of on-campus and 50% of off-campus residents were FI, and 70% of FI students reported needing help accessing food. Cooking was undertaken “less often” by 61.5% of FS and 55.8% of FI sophomores. Mean cooking self-efficacy scores for FS and FI students were 44.9, vs 43.4, (p > 0.05) out of 52 points. Grains were consumed most often by 40% of FS and FI students and vegetables were consumed least often by 70% of both groups. Mean food safety test scores for FS and FI students were 6.2 1.60 vs 6.6 1.52 (p > 0.05) out of 11 points. Requested educational activities included making a budget and planning balanced meals.

**Implications:**

The high rate of food insecurity reflects an ongoing need among sophomores for campus and community food assistance and for educational activities that teach purchasing and preparation of affordable, healthy and safe foods.

## INTRODUCTION

Food insecurity means lacking regular access, in socially acceptable ways, to an adequate, nutritious, safe diet that promotes an active and healthy life.^1^ The U.S. Department of Agriculture (USDA) administers the Adult Food Security Survey (AFSS) annually^2^ to measure the food security status of the adult population. Respondents are classified along a continuum from high to very low food secure. Questions assess the quantity, quality, variety, and desirability of their available food supply. In 2020, the rate of food insecurity (combined low and very low) was 10.5%, and that of very low food security was 3.9%.^3^ Groups with food insecurity rates exceeding the national average were households with children, single parents, men and women living alone, black, non-Hispanics, Hispanics, those with incomes below 185% of the poverty threshold, those living in nonmetropolitan areas, and those residing in the South.^3^ The unfavorable health outcomes associated with prolonged food insecurity include obesity and the metabolic syndrome,^4^ mental health disorders,^5^ cognitive decline,^6^ and poor growth and development.^7^

Ample evidence from 2- and 4-year public and private postsecondary institutions nationwide has identified college students as a vulnerable group for food insecurity,^8^ with rates ranging from 14.8% at an urban university in Alabama^9^ to 59.0% at a rural university in Oregon.^10^ The present study was conducted at a mid-sized university in Appalachia, where in 2016 the rate of student food insecurity was 46.2%.^11^ Rates previously reported for ten postsecondary institutions in the Appalachian and southeastern regions ranged from 22.4% to 51.8%.^12^ The sociodemographic characteristics most frequently associated with food insecure (FI) college students include: older age, identifying with a minority race/ethnic group, receiving food assistance, having less money to buy food, being employed while in school,^13^ and low grade point average (GPA).^14^ Additionally, when compared to their food secure (FS) peers, FI students show higher rates of overweight and obesity^15^ and of gastrointestinal, neurologic, and mental health disorders.^16^

In addition to measuring prevalence rates of campus food insecurity and identifying associated characteristics, researchers have also assessed the food preparation behaviors, dietary practices, and diet quality of FI college students, key determinants of food security.^1^ Food preparation behaviors include stretching food to make it last longer,^11^ low rates of cooking for self or others,^9^ and low cooking self-efficacy.^17^ Findings concerning dietary practices and diet quality include meal skipping and eating less healthy meals to eat more,^11^ and lower consumption of fruits and higher consumption of added sugars,^18^ lower consumption of vegetables and legumes,^19^ and overeating when food is plentiful.^20^ Literature searches in PubMed and ScienceDirect located no study concerning the third determinant of food security, i.e., access to safe food.^1^ Investigators assessing the food safety knowledge among campus-wide samples have reported low awareness about safe food purchasing, storage, and preparation.^21^,^22^

The aim of the present study was to compare FS and FI sophomores attending a university in Appalachia on cooking, dietary, and food safety characteristics related to the three USDA determinants of food security, i.e., regular access to an adequate, nutritious, and safe diet.^1^ Recruitment was restricted to sophomores because transitioning from campus to community housing is common at the study site,^23^ and research is needed to establish baseline characteristics that affect food security status in this potentially vulnerable group.^24^

## METHODS

### Participants and Recruitment

Recruitment was accomplished using a non-probability, randomized, computer-generated list of email addresses for 1794 sophomores provided by the university. Inclusion criteria were at least 18 years of age, any gender identity, on or off-campus residence, and any race/ethnic affiliation. Recruitment began during mid-February 2019 with four weekly email reminders.^25^ This time was chosen to obtain a more accurate measure of the students’ food security status by avoiding the first four weeks of the semester when they may have had access to food resources obtained during the winter break. The students clicked a link in the recruitment email that directed them to a screen displaying the elements of informed consent. Those wishing to participate clicked on a “yes” button that displayed the questionnaire. Students wishing to enter a drawing for a $50 gift card from Amazon.com clicked a link taking them to a detached Qualtrics survey where they entered their email address. This study was exempt by the Office of Research Protections at Appalachian State University (study number 19-0172).

### Questionnaire

Data were collected using an anonymous online questionnaire administered through Qualtrics (Qualtrics, Provo UT; August 2019). The questionnaire contained 49 close-ended items and took approximately 15 minutes to complete. Content validity was confirmed by three nutrition professors familiar with the college student food insecurity literature and with experience at questionnaire design. The initial draft was pilot tested online with 5 sophomores who did not participate in the final study. No problems were identified with wording of items, functionality of buttons, or screen displays.

### Measures

The dependent variable was food security status, and the independent variables were cooking frequency and cooking self-efficacy (pertinent to regular access to an adequate diet),^26^ food group consumption (pertinent to a nutritious diet),^27^ food safety knowledge and safe food handling (pertinent to a safe diet),^28^ need for social support accessing food, and educational activities to improve food access. “Food access” includes food utilization, which refers to the uses of food in the home, such as food distribution and food preparation.^26^

#### Food security status

The dependent variable was measured using the 10-item USDA AFSS,^2^ and scores were determined by allotting 1 point to each affirmative response, i.e., “yes,” “often,” “sometimes,” “almost every month,” and “some months but not every month.” Students were assigned to a food security category as follows: high (0 [zero]), marginal (1–2), low (3–5), or very low (6–10). Students in the high and marginal categories were classified as food secure (FS) and those in the low and very low categories as food insecure (FI).^2^

#### Cooking frequency

This behavior was estimated by having the students select either “never,” “< once/week,” “once/day,” “2–3 times/day,” or “4+ times/day.” Data were analyzed by compressing the responses into a “less often” category (never, < once/week, and once/day) and a “more often” category (2–3 times/day and 4+ times/day).

#### Cooking self-efficacy

This variable was measured with a list of ten food preparation and three safe food handling activities. Scores were determined by allotting 1 point to “not at all confident”; 2 to “a little confident”; 3 to “confident”; and 4 to “very confident,” with possible scores ranging from 13 to 52. This scale was developed with guidance from research conducted by Laska et al.^29^ concerning the cooking skills of adolescents.

#### Food group consumption

Students estimated how often they consumed foods from the five food groups on the USDA MyPlate graphic^27^ and from a sweets group by responding to the question: “About how many times per day do you eat from each of the following food groups?” with the answer choices 0 (zero), 1–2, 3–4, 5–6, or 7+ times/day. Data were analyzed by compressing the responses into a “least often” category (0 [zero] and 1–2 times/day) and a “most often” category (3–4, 5–6, and 7+ times/day). Additionally, the students were asked to “Check the food group(s) that you would eat more from if you had greater access…” from a list of the five food groups and a sweets group. Frequency counts and percentages were calculated, and the findings were ranked in descending order.

#### Food safety knowledge

The students completed an 11-item multiple-choice food safety test, with each question followed by four answer choices. The test addressed food characteristics (2 questions), storage (3 questions), preparation (3 questions), and risk reduction (3 questions). Scores were calculated by allotting 1 point to correct and 0 (zero) points to incorrect responses, with possible scores ranging from 0 to 11. This test was constructed with guidance from research conducted by Green and Knechtges^21^ and by McNeilly and Raming.^22^

#### Social support and educational activities for food access

The students indicated how much support they could have used to help them access food by responding to the statement: “I could use _____ support accessing food” with the answer choices “a lot,” “some,” “a little,” or “I do not need help.” Those who needed some level of support checked, from the following list of educational activities, those they believed would have helped them improve their access to food during their sophomore year: make a budget, container gardening, community gardening, plan balanced meals, make a grocery list, purchase affordable, healthy foods, use different cooking skills, and shop for, store, and prepare foods safely. These educational activities were compiled from studies that asked students to identify types of learning opportunities that they thought might improve their food access.^9^,^11^,^12^,^30^,^31^ Responses were tallied and reported in descending order.

#### Sociodemographic and health measures

The sociodemographic questions asked for information about gender identity, ethnicity, living arrangement, participation in campus meal plan, financial aid status, employment status, personal monthly income, and annual family income. Health-related questions asked for self-reported height and weight and self-rated physical and mental/emotional health. Students rated their physical and mental/emotional health by responding to the questions, “I would rate my current physical health as. . .” and “I would rate my current mental/emotional health as. . .” with the response options “poor,” “fair,” “good,” or “very good.”

### Data Analysis

Data were analyzed using the Statistical Package for the Social Sciences (SPSS Statistics for Windows, Version 24, IBM Corp., Armonk NY, 2016). Frequency counts and percentages were obtained on the dependent and independent variables. Chi-square analyses compared percentages of FS and FI students on frequency of food group consumption, correct answers on the food safety test, and self-rated physical and mental/emotional health. T-tests compared mean scores of FS and FI students on the cooking self-efficacy scale and on the food safety test. Multi-variable modeling was used to relate food insecurity to the independent variables (cooking frequency, cooking self-efficacy, food group consumption, food safety knowledge, need for social support for accessing food, and educational activities regarded as helpful for improving food access) while controlling for possible confounding demographic variables (gender, race/ethnicity, BMI category, self-rated physical health, self-rated mental/emotional health, receiving financial aid, personal monthly income, and annual family income). Both multiple linear regression (with AFSS score as the dependent variable) and logistic regression (to model the probability of food insecurity) were used to examine this relationship (p<0.05).

## RESULTS

### Participant Profile

Questionnaires were submitted by 242 sophomores; 16 were discarded due to incomplete AFSS data, yielding a sample of 226 participants (12.5% of those recruited). The students were 65.0% females and 76.1% non-Hispanic whites, with a mean age of 19.5 years (±1.2, range 18 to 29). Comparisons with the overall sophomore enrollment indicated that the sample overrepresented white, non-Hispanic and female students.

School-related findings revealed that 88.1% of the sophomores were full-time students, 42.5% lived on-campus, and 51.8% participated in a campus meal plan. Regarding socioeconomic characteristics, 49.1% of the sophomores were financial aid recipients, 42.3% were employed while in school, and 22.6% selected the lower, 34.5% the middle, and 31.9% the upper annual family income category.

### Characteristics of Food Secure and Food Insecure Sophomores

The sophomores were 54% FS (n = 122) and 46% FI (n = 104). Table 1 compares frequency distributions of characteristics among FS and FI sophomores.

In summary, gender had a significant effect on the students’ food security status (p=0.036), with a greater percentage of males (60%) than females (54%) in the FS group. Race/ethnicity approached but did not reach significance, with about 60% of the white, non-Hispanic students in the FS group and about 60% of the non-white students in the FI group.

Health-related findings indicated that BMI category, based on self-reported heights and weights, had a significant effect on food security status (p=0.029), with a majority (60%) of the underweight/normal weight students in the FS group and a majority (57%) of the overweight/obese students in the FI group. Among students who rated their physical health as “good” or “very good,” about two-thirds were FS, whereas among those rating their physical health as “poor” or “very poor,” about two-thirds were FI (p<0.001). A similar pattern of self-ratings was observed for the mental/emotional health classification (p=0.001) between FS and FI students.

Among full-time students, approximately 56% were FS and 44% were FI, while two-thirds of the part-time students were FI. Sixty percent of the on-campus residents were FS and 40% were FI; for off-campus residents 50% were FS and 50% were FI. Since 20 students did not disclose their living arrangement (nine FS and 11 FI), it could not be determined if any of the 11 FI nondisclosures were also homeless.

Socioeconomic findings indicated that about 60% of the students who participated in a campus meal plan were FS and 40% were FI, approximately 47% of those who received financial aid were FS and 52% were FI (p=0.013), and about 52% of employed students were FS and 48% were FI. The findings concerning the students’ personal monthly income indicated that in each of the three income categories (lower, middle, and upper), about 55% were FS and 45% were FI. Annual family income had a significant effect on the students’ food security status (p=0.003). The percentage of FS students increased from 37% in the lower family income category to 68% in the upper family income category.

### Cooking Frequency and Cooking Self-Efficacy

No significant differences were found between FS and FI students in the frequencies with which they cooked for themselves regardless of on or off-campus residences; 61.5% of the FS and 55.8% of the FI students cooked for themselves less often. Likewise, no significant difference was found between the mean scores of the two groups on the 13-item cooking self-efficacy scale (FS = 44.9 ±7.2, range 26 to 52 vs FI = 43.4 ±7.1, range 21 to 52 out of a possible 52 points). Table 2 shows the mean scores of the FS and FI students on these items.

Findings revealed that, although the FS students earned higher mean scores on all cooking activities, the two groups differed significantly (p<0.001) only on “cooking foods using the microwave” and “accurately using measuring cups and spoons,” with the FS students earning higher scores. The two activities reflecting the lowest self-efficacy for the FS and FI students were “using leftovers” and “cooking new foods,” while the two reflecting the highest self-efficacy were “using the microwave” and “accurately using measuring cups and spoons.”

### Food Group Consumption

Table 3 shows frequencies of food group consumption by the FS and FI sophomores.

Grain and cereal products and protein foods were consumed most often by about 40% of the FS students and fruits and fruit juices by 35%, while grain and cereal products and dairy foods were consumed most often by about 40% of the FI students and protein foods by 35%. Sweets were consumed least often by 75% of the FS students, vegetables and vegetable juices by 70%, and dairy foods by 65%, while vegetables and vegetable juices, fruits and fruit juices, and sweets were consumed least often by 70% of the FI students. The following food groups were identified by the FS and FI students, respectively, as those they would consume more from if given greater access: fruits and fruit juices (61.5% vs 79.8%, p<0.01), vegetables and vegetable juices (60.7% vs 73.1%, p<0.05), protein foods (34.4% vs 52.9%, p<0.01), and dairy foods (13.1% vs 25.0%, p<0.05). No significant differences were found for these variables between on and off-campus residents.

### Food Safety Knowledge

There were no significant differences between the mean scores of the FS and FI sophomores, respectively, on the 11-item food safety knowledge test (6.2 ±1.6 vs 6.6 ±1.5). Likewise, no significant differences were found between their mean scores on the four categories of questions, i.e., food characteristics, safe storage, safe food preparation, and risk reduction.

Table 4 shows frequency distributions of correct answers from the FS and FI sophomores.

The question most frequently answered correctly by both groups was “Which food is most likely to become contaminated with bacteria that cause foodborne illness?” from the food characteristics category, and the question least often answered correctly was “At what temperature should you keep your freezer to store food safely?” from the safe food storage category. These test scores showed no significant differences between on or off-campus residents.

### Social Support and Educational Activities for Food Access

The FS and FI sophomores, respectively, indicated that they could have used the following levels of help accessing food during their sophomore year: “a lot” 0.0% vs 8.7%, “some” 4.1% vs 22.1%, “a little” 13.1% vs 40.4%, and “I do not need help” 82.8% vs 28.8%, all p<0.001.

The educational activities that the FS and FI sophomores, respectively, believed would have helped improve their food access were those that taught how to: shop for affordable healthy foods (13.1% vs 50.0%), plan balanced meals (13.1% vs 46.2%), make a budget (11.5% vs 39.4%), and handle food safely (6.6% vs 24.0%), all p<0.001.

### Multi-Variable Modeling

Since the multiple linear regression and logistic regression models provided essentially the same conclusion, only the results of the former are presented. In the multiple linear regression model relating AFSS score to the independent variables while controlling for possible confounding demographic variables, the demographic variables alone explained 21% of the variation in AFSS score. After controlling for potential confounders, the significant explanatory variables were (1) the need for social support when accessing food (p<0.001, increased R-square from 21% to 38%), and (2) several of the educational activities for improving food access: make a budget, community gardening, plan balanced meals, make a grocery list, and purchase affordable, healthy foods. These were all significant at the p<0.001 level, and as a group increased the R-square from 21% to 38%. When modeled together, the need for social support variable and the five educational activities increased the R-square from 21% (demographic confounders alone) to 47% for the full model.

## DISCUSSION

Nearly half (46%) of the sophomores in this study were FI based on their AFSS scores, and about 70% of these students indicated that they could have used some level of help accessing food. Additionally, 40% or more of the students who had access to some food through participation in a campus meal plan, and who had access to some monetary resources from financial aid and employment wages were, nevertheless, FI. Since need for social support accessing food was a primary explanatory variable for the AFSS scores, more food and financial resources would have been needed to improve the problem of food insufficiency among these participants.

In addition to the challenge of regular food access, the FI sophomores also reported lower than recommended frequencies of food group consumption (i.e., fruits, vegetables, dairy)^27^ and earned low scores on the food safety test. Collectively, these findings suggest that the cohort of FI sophomores did not meet the three determinants in the USDA definition of food security,^1^ despite the presence of several campus food pantries.^32^ Several factors may have contributed to limited food access by these students. Notably, food insecurity was more prevalent among the sophomores who transitioned to community housing than among the on-campus residents. This finding suggests that, for some students, relocating may have meant new and recurring financial challenges such as rent, groceries, utilities, transportation, and internet costs. Under circumstances of restricted funds, these expenses, in addition to tuition and other school fees, may have curtailed funds for food purchasing, especially among students with limited budgeting skills, as reported by Gaines et al.^9^ for FI students at a university in Alabama. Making a budget was one of the educational activities that the students in the present study believed would help them improve their food access, suggesting that they would be receptive to such instruction. Findings also indicated that annual family income had a significant effect on the students’ food security status, i.e., as family income increased, the percentage of FI sophomores decreased. We speculate, therefore, that the students from wealthier families may have received more food and financial assistance to purchase food than the students from the lower income categories, which showed greater percentages of FI students. We acknowledge, however, that access to food resources from home was not examined in this study.

More than half of the FS and FI sophomores cooked either “never,” “less than once a week,” or “once a day,” corroborating the findings of Hagedorn et al.^12^ and of Knol et al.^17^ for FI students. A possible explanation for the low cooking frequency among the FI sophomores who participated in a campus meal plan may be that they preferred to use their meal cards to buy meals and snacks at dining halls rather than to spend more money on food and ingredients for cooking. The on-campus residents in particular may have found it challenging to locate facilities where they could cook, since access to kitchens and cooking appliances are not readily available in residence halls. Regarding the FI students who relocated to community housing, while kitchens and basic cooking appliances would likely be available in apartments and houses, monthly bills may nevertheless have restricted their food budget, limiting cooking opportunities. Time constraints may also have contributed to the low cooking frequency reported by FS and FI students. Despite their less frequent cooking behaviors, both groups earned above average mean scores on the cooking self-efficacy scale, in contrast to findings reported by Gaines et al.^9^ and Knol et al.^17^ Possibly these students felt confident in their cooking abilities because they had more opportunities to cook and to strengthen their cooking skills during holiday and summer breaks when they had more time, more money from jobs to purchase food, or greater access to family food resources. The students’ cooking frequency when not in school was not investigated.

Food group consumption by the FS and FI sophomores was similar and revealed that neither group met MyPlate recommendations.^27^ Similar dietary patterns were reported by Oo et al.^33^ for FI students at the University of Kentucky. Such suboptimal dietary patterns in the long run, as over the course of a 4-year undergraduate career, could compromise the students’ nutrient reserves,^15^,^18^,^19^ physical health,^4^,^15^,^16^,^20^,^34^ mental and emotional health,^14^,^16^,^35^ and academic success.^12^,^19^,^35^ Frequent consumption of grain and cereal products and protein foods, such as that reported by the sophomores in this study, is associated with diets high in simple carbohydrates and saturated and trans fats. Several authors have reported that FI students tend to choose such nutrient-poor and low-cost foods as a coping mechanism to feel full,^18^,^19^ increasing their risk for obesity and related cardiometabolic conditions.^20^,^34^ In this regard, a significantly greater percentage of sophomores who self-reported being overweight or obese were FI. Given that food group consumption was similar for the FS and FI sophomores, the higher occurrence of excess adiposity among the FI students could be attributable to variables not measured in this study, such as portion sizes consumed, regular consumption of fast foods, and sedentary lifestyles. Other health-related findings were that significantly greater percentages of students who rated their physical and their mental/emotional health as “poor” or “fair” were FI, as reported by other investigators.^14^,^35^

The FS and FI students identified educational activities that teach food purchasing, preparation, budgeting, production, and safe food handling skills as those they believed would enhance their food access. Such activities could encourage greater adherence to MyPlate recommendations^27^ and bolster the students’ nutrient reserves. Activities should emphasize the health-promoting benefits of fruits, vegetables, and calcium-rich foods, along with budget-friendly strategies to incorporate them in the diet. These foods were consumed least often, and the students indicated that such foods would be consumed more often given greater access. Additionally, offering safe food handling participatory activities could enhance the students’ food supply by reducing food waste. These activities could serve as the core for an interdisciplinary food security skill-building course. Such a course was taught at the study site during the spring, 2019 semester, and reflection essays indicated that the students found the skills taught helpful, especially the instruction on budget preparation and healthy meal planning.

Other recommendations for administrators to reduce the high rate of campus food insecurity include redistributing unused meal card funds, alerting students when free food from catered events is available, and offering food scholarships to FI students.^30^ Additionally, promotion of the campus food pantries remains an important temporary strategy for decreasing campus food insecurity. In addition to offering a variety of prepackaged and fresh foods, spices, small cooking appliances, and cooking utensils, the pantries also offer recipes for preparing healthy and affordable foods and assist students in applying for SNAP benefits. These facilities could also serve as a classroom for teaching some of the skills identified by the students as those they believed would improve their long-term food access, such as making a budget, planning meals, and shopping for affordable foods. Validating these types of educational activities to determine their effectiveness for reducing campus food insecurity is needed. In this regard, positive outcomes, including a 22% decrease in food insecurity, were reported by Matias et al.^31^ from a one semester college course that provided instruction about budgeting, meal preparation, basic nutrition, and food safety using a teaching kitchen.

## IMPLICATIONS

The high rate of food insecurity among the sophomores in this study reflects an ongoing need for campus, state, and federal policies and programs that facilitate greater access to food by this vulnerable cohort of college students, and for educational activities that teach the types of skills the students regard as helpful for improving their food access, i.e., food budgeting, preparing affordable, healthy foods, and safe food handling. Several limitations prevent the generalizability of the findings to sophomores at other Appalachian campuses, including a modest sample size, overrepresentation of females and whites, and self-reporting of all data. Additionally, although the food safety test and cooking self-efficacy scales were rooted in pertinent literature, these assessment instruments should be validated with college students in future studies. The higher rate of food insecurity among the off-campus cohort provides opportunities for continued research on factors associated with community living that may be contributing to this problem. Future research could also investigate the specific food and financial supports that are most impactful in reducing student food insecurity given that social support was an important explanatory variable for food security status. Lastly, since direct observation of cooking skills and food handling was not performed, research is warranted to identify gaps in these behaviors that could serve as the basis for educational activities for increasing the students’ access to an adequate, nutritious, and safe diet, in line with the USDA definition of food security.^1^

**SUMMARY BOX**

**What is already known on this topic?**

High rates of collegiate food insecurity have been reported on ten Appalachian campuses.

**What is added by this report?**

Transitioning to community housing impacted the students’ food security status; greater percentages of off-campus sophomores were FI compared to their on-campus peers. The students’ limited access to food as reflected in their AFSS scores, inadequate food group intake, and low food safety scores suggest that about half the sophomores in this study did not meet the USDA criteria for food security. The need for social support was a primary explanatory characteristic for students’ food security status.

**What are the implications for future research?**

Studies are needed that identify predisposing factors for food insecurity among sophomores who relocate to community housing. Observational research is also warranted that identifies gaps in food purchasing, preparation, and handling skills to design activities for enhancing the students’ food supply. Future research should also investigate the specific food and financial supports that are most impactful in reducing student food insecurity.

## Figures and Tables

**Table 1:** Frequency Distributions of Characteristics Among Food Secure (*n* = 122) and Food Insecure (*n* = 104) Sophomores

	Food Secure Students		Food Insecure Students		
Characteristic	*n*	%	*n*	%	p-value
**Gender**					
Males	33	60.0	22	40.0	
Females	80	54.4	67	45.6	0.036
Nonbinary	0	0.0	5	100.0	
Missing	9	47.4	10	52.6	
**Ethnicity**					
White, Non-Hispanic	99	57.6	73	42.4	
Non-White	14	40.0	21	60.0	0.057
Missing	9	47.4	10	52.6	
**Weight category by BMI**					
Underweight/Normal weight (18.5–24.9 kg/m2)	83	60.1	55	39.9	
Overweight/Obese (25.0–30.0 kg/m2)	27	43.5	35	56.5	0.029
Missing	12	46.2	14	53.8	
**Self-Rated physical health**					
Poor/Fair	25	34.2	48	65.8	
Good/Very good	95	65.5	50	34.5	< 0.001
Missing	2	25.0	6	75.0	
**Self-Rated mental/emotional health**					
Poor/Fair	42	42.4	57	57.6	
Good/Very good	78	65.5	41	34.5	0.001
Missing	2	25.0	6	75.0	
**Academic status**					
Part-time	1	33.3	2	66.7	
Full-time	112	56.3	87	43.7	0.427
Missing	9	37.5	15	62.5	
**Living arrangement**					
On campus	58	60.4	38	39.6	
Off campus	55	50.0	55	50.0	0.134
Homeless	0	0.0	0	0.0	
Missing	9	45.0	11	55.0	
**Campus meal plan**					
Yes	70	59.8	47	40.2	
No	50	49.5	51	50.5	0.127
Missing	2	25.0	6	75.0	
**Financial aid**					
Yes	52	46.8	59	53.2	
No	61	64.2	34	35.8	0.013
Missing	9	45.0	11	55.0	
**Employment**					
Employed	50	52.1	46	47.9	
Unemployed	63	56.8	48	43.2	0.501
Missing	9	47.4	10	52.6	
**Personal monthly income**					
Lower ($0 – $500)	96	54.9	79	45.1	
Middle ($501 – $1000)	11	55.0	9	45.0	1.000
Upper ($1000+)	6	54.5	5	45.5	
Missing	9	54.9	11	45.1	
**Annual family income**					
Lower ($0 – $34,999)	19	37.3	32	62.7	
Middle ($35,000 – $99,999)	41	52.6	37	47.4	0.003
Upper ($100,000 – $200,000+)	49	68.1	23	31.9	
Missing	13	52.0	12	48.0	

*Note:* Food secure is defined as having either high or marginal food security (scores of 0 [zero] to 2) and food insecure is defined as having either low or very low food security (scores from 3 to 10) on the USDA Adult Food Security Survey (AFSS).

**Table 2:** Mean Self-Efficacy Cooking and Food Handling Scores of Food Secure *(n* = 122*)* and Food Insecure *(n* = 104*)* Sophomores

	Food Secure Students	Food Insecure Students	p-value
Activity	Mean (±SD)*	Mean (±SD)*	
Cooking foods using the microwave	3.80 (±0.45)	3.66 (±0.56)	< 0.001
Accurately using measuring cups and spoons	3.74 (±0.53)	3.55 (±0.73)	< 0.001
Accurately setting temperatures on the stove and oven	3.73 (±0.53)	3.70 (±0.48)	0.873
Using a cutting board	3.67 (±0.63)	3.64 (±0.60)	0.931
Following a simple recipe	3.61 (±0.64)	3.56 (±0.66)	0.420
Storing cold and frozen foods safely	3.45 (±0.76)	3.40 (±0.72)	0.406
Using a blender	3.43 (±0.83)	3.36 (±0.82)	0.742
Using knives to slice, chop, dice, or mince	3.43 (±0.80)	3.35 (±0.85)	0.438
Preparing foods safely	3.40 (±0.74)	3.27 (±0.72)	0.694
Making safe food purchases	3.33 (±0.85)	3.11 (±0.89)	0.954
Preparing meals that include vegetables	3.27 (±0.91)	3.12 (±0.90)	0.224
Using leftovers to make different foods	3.10 (±1.02)	2.86 (±1.06)	0.455
Cooking new foods	2.96 (±0.95)	2.86 (±0.99)	0.324

* Scores were determined by allotting points according to confidence level: not at all (1), a little confident (2), confident (3), very confident (4).

*Note:* Food secure is defined as having either high or marginal food security (scores of 0 [zero] to 2) and food insecure is defined as having either low or very low food security (scores from 3 to 10) on the USDA Adult Food Security Survey (AFSS).

**Table 3:** Frequency of Food Group Consumption by Food Secure *(n* = 122*)* and Food Insecure *(n* = 104*)* Sophomores

	Food Secure Students				Food Insecure Students			
	Most Often*		Least Often		Most Often*		Least Often	
Food Group	*n*	%	*n*	%	*n*	%	*n*	%
Grains/cereals	52	42.6	67	54.9	45	43.3	51	49.0
Vegetables and juices	31	25.4	88	72.1	21	20.2	75	72.1
Fruits and juices	42	34.4	77	63.1	23	22.1	73	70.2
Meat, seafood, and poultry	41	33.6	78	63.9	35	33.7	61	58.7
Other protein foods	51	41.8	67	54.9	36	34.6	58	55.8
Dairy foods	39	32.0	80	65.6	40	38.5	56	53.8
Sweets	26	21.3	93	76.2	24	23.1	72	69.2

* Most often was defined as 3, 4, 5, 6, or 7 or more times/day; least often was defined as 0 (zero), 1, or 2 times/day.

**Table 4:** Frequency Distributions of Correct Answers from Food Secure (*n* = 122) and Food Insecure (*n* = 104) Sophomores on the Food Safety Knowledge Test

Topic	Answer Choices	Food Secure		Food Insecure	
		*n*	%	*n*	%
Food Characteristic					
Which food is most likely to become contaminated with bacteria that cause foodborne illness?	Chicken^*^ Black beans Bread Baked potatoes	111	91.0	93	89.4
Which characteristic of food is associated with an increased risk of foodborne illness?	Low-sugar High-protein^*^ High-acid Low-moisture	54	44.3	53	51.0
Safe Storage					
What is the longest time leftover turkey can be safely left on the table?	4 hours 30 minutes 2 hours^*^ 1 hour	36	29.5	36	34.6
At what temperature should you keep your freezer to store food safely?	0° F^*^ 15° F 25° F 32° F	20	16.4	20	19.2
Where should fresh meats be placed in your refrigerator?	With produce On the top shelf On the bottom shelf^*^ They should not be stored in your refrigerator	85	69.7	70	67.3
Safe Food Preparation					
Which is the safe temperature for reheating meat and poultry?	75° F 120° F 100° F 165° F^*^	66	54.1	54	51.9
Which food is being thawed improperly?	Whole chicken thawed in a refrigerator Frozen fish thawed under cool running water Frozen turkey thawed on the kitchen counter at room temperature^*^ Frozen hamburger patties thawed on a grill while they are being cooked	37	30.3	31	29.8
Which best describes the appearance of a hamburger when it is safely cooked?	Mostly pink on the inside Brown all the way through^*^ Some pink on the inside Some pink on the outside	59	48.4	56	43.8
Risk Reduction					
Which is an important strategy for reducing your risk of foodborne illness?	Wash hands before and after handling raw meat, poultry, fish, or shellfish Cook and reheat foods at the temperatures shown in the recipe or on the package Refrigerate leftovers immediately after serving All of the above^*^	111	91.0	92	88.5
How long should you spend washing your hands with soap and warm water before and after preparing food?	10 seconds 20 seconds^*^ 2 minutes 1 minute	34	27.9	34	32.7
Which product would be safe to buy?	A carton with one cracked egg Yogurt with a past expiration date A punctured can of green beans A frozen pizza with no ice crystals on the package^*^	91	74.6	83	79.8
Correct answers are identified with a ^*^ symbol.					

*Note:* Food secure is defined as having either high or marginal food security (scores of 0 [zero] to 2) and food insecure is defined as having either low or very low food security (scores from 3 to 10) on the USDA Adult Food Security Survey (AFSS).
